# Latent Growth Mixture Models to estimate PTSD trajectories

**DOI:** 10.3402/ejpt.v6.27503

**Published:** 2015-03-02

**Authors:** Rens Van de Schoot

**Affiliations:** Department of Methods and Statistics, Utrecht University, Utrecht, The Netherlands. Email: a.g.j.vandeschoot@uu.nl; Optentia Research Program, Faculty of Humanities, North-West University, Vanderbijlpark, South Africa

Statistical models to estimate individual change over time and to investigate the existence of latent trajectories, where individuals belong to trajectories that are unobserved (latent), are becoming ever more popular. Such models are called Latent Growth Mixture Models (LGMM; Muthén & Muthén, 2000) and are often applied to estimate posttraumatic stress (PTSD) trajectories across several months/years following a traumatic event (Armour, Shevlin, Elklit, & Mroczek, 2012; Berntsen et al., 2012; Bonanno et al., 2012; Forbes et al., 2010; Galatzer-Levy et al., 2013; Mouthaan et al., 2013; Van de Schoot, Broere, Perryck, Zondervan-Zwijnenburg, & Van Loey, 2015; Van Loey, Van de Schoot, & Faber, 2012). The purpose of LGMM is to search for “hidden” subpopulations that are characterized by a different developmental process (growth trajectory). With LGMM it is hypothesized that there are different latent classes each with their own growth model.

Supported by a grant from the Netherlands Organization for Scientific Research, an international meeting was organized to present the current state of affairs concerning LGMM to investigate the causes and consequences of PTSD. Three key aspects of LGMM and its application in the field of psychotrauma were presented in the old University Hall at Utrecht University (founded in 1462), The Netherlands. The first presentation introduced LGMM and provided guidelines on which models to run, how to interpret the results, and what to report in a paper (Van de Schoot, 2015). The second presentation discussed the current state of affairs in applying LGMM models to PTSD data (Galatzer-Levy, 2015). The last presentation demonstrated that only the Bayesian approach results in a theory-driven solution of estimating the delayed onset trajectory (Depaoli, Van de Schoot, Van Loey, & Sijbrandij, 2015).

The meeting was endorsed by the International Society for Traumatic Stress Studies (ISTSS), and part of the ISTSS global meetings program. “We are excited about new and advanced statistical techniques, in particular Bayesian LGMM, since these can answer new research questions and deal with commonly encountered problems like having to deal with small data sets” (Olff, 2015).

*Rens Van de Schoot* Department of Methods and Statistics Utrecht University Utrecht, The Netherlands Email: a.g.j.vandeschoot@uu.nl Optentia Research Program, Faculty of Humanities North-West University Vanderbijlpark, South Africa
